# The Preventive Effects of Xanthohumol on Vascular Calcification Induced by Vitamin D_3_ Plus Nicotine

**DOI:** 10.3390/antiox9100956

**Published:** 2020-10-06

**Authors:** Shu-Fen Liou, Thi Tuyet Ngan Nguyen, Jong-Hau Hsu, Erna Sulistyowati, Shang-En Huang, Bin-Nan Wu, Ming-Chung Lin, Jwu-Lai Yeh

**Affiliations:** 1 Department of Pharmacy, Chia-Nan University of Pharmacy and Science, Tainan 717, Taiwan; shufen2139@mail.cnu.edu.tw; 2 Graduate Institute of Medicine, College of Medicine, Kaohsiung Medical University, Kaohsiung 807, Taiwan; tuyetngan9296@gmail.com (T.T.N.N.); jhh936@yahoo.com.tw (J.-H.H.); dr_erna@unisma.ac.id (E.S.); eva_1433@yahoo.com.tw (S.-E.H.); binnan@kmu.edu.tw (B.-N.W.); 3 Department of Pediatrics, Kaohsiung Medical University Hospital, Kaohsiung 807, Taiwan; 4 Department of Pediatrics, College of Medicine, Kaohsiung Medical University, Kaohsiung 807, Taiwan; 5 Faculty of Medicine, University of Islam Malang, Malang city, East Java 65145, Indonesia; 6 Department of Pharmacology, College of Medicine, Kaohsiung Medical University, Kaohsiung 807, Taiwan; 7 Department of Medical Research, Kaohsiung Medical University Hospital, Kaohsiung 807, Taiwan; 8 Department of Anesthesiology, Chi Mei Medical Center, Tainan 710, Taiwan; 9 Department of Marine Biotechnology and Resources, National Sun Yat-sen University, Kaohsiung 804, Taiwan

**Keywords:** xanthohumol, vascular calcification, vitamin D_3_, nicotine, osteogenic transition, oxidative stress

## Abstract

Vascular calcification (VC) is highly prevalent in patients with atherosclerosis, chronic kidney disease, diabetes mellitus, and hypertension. In blood vessels, VC is associated with major adverse cardiovascular events. Xanthohumol (XN), a main prenylated chalcone found in hops, has antioxidant effects to inhibit VC. This study aimed to investigate whether XN attenuates VC through in vivo study. A rat VC model was established by four weeks oral administration of vitamin D_3_ plus nicotine in Sprague Dawley (SD) rats. In brief, 30 male SD rats were randomly divided into three groups: control, 25 mg/kg nicotine in 5 mL corn oil and 3 × 10^5^ IU/kg vitamin D_3_ administration (VDN), and combination of VDN with 20 mg/L in 0.1% ethanol of XN (treatment group). Physiological variables such as body and heart weight and drinking consumption were weekly observed, and treatment with XN caused no differences among the groups. In comparison with the control group, calcium content and alkaline phosphatase (ALP) activity were increased in calcified arteries, and XN treatment reduced these levels. Dihydroethidium (DHE) and 2′,7′-dichloroflurescin diacetate (DCFH-DA) staining to identify Superoxide and reactive oxygen species generation from aorta tissue showed increased production in VDN group compared with the control and treatment groups. Hematoxylin eosin (HE) and Alizarin Red S staining were determined to show medial vascular thickness and calcification of vessel wall. Administration of VDN resulted in VC, and XN treatment showed improvement in vascular structure. Moreover, overexpression of osteogenic transcription factors bone morphogenetic protein 2 (BMP-2) and runt-related transcription factor 2 (Runx2) were significantly suppressed by XN treatment in VC. Moreover, downregulation of vascular phenotypic markers alpha-smooth muscle actin (α-SMA) and smooth muscle 22 alpha (SM22α) were increased by XN treatment in VC. Furthermore, XN treatment in VC upregulated nuclear translocation of nuclear factor-E2-related factor 2 (Nrf2) and heme oxygenase-1 (HO-1) expressions. Otherwise, Kelch-like ECH-associated protein 1 (Keap1) was alleviated by XN treatment in VC. In conclusion, our findings suggested that XN enhances antioxidant capacity to improve VC by regulating the Nrf2/Keap1/HO-1 pathway. Therefore, XN may have potential effects to decrease cardiovascular risk by reducing VC.

## 1. Introduction

Vascular calcification (VC) is a perplexing clinical concern, and a major risk factor for many cardiovascular diseases, especially for pathology in aging, atherosclerosis, hypertension, and diabetes. Moreover, it correlates with vessel stiffening and increased risk of myocardial infarction. The calcium-phosphorus deposition contributes to structural damage and fibrosis proliferation and formation of calcium nodules in the middle layer of artery, which can result in the thickening of arterial walls and reducing of vascular compliance [1]. Vascular smooth muscle cells (VSMC) are involved in the different stages of lesion development. Accumulating studies have demonstrated that VC is highly regulated by biological processes involving the transformation from the VSMC’s phenotype into osteoblast-like cells. VC is characterized by decreased expression of contractile proteins including smooth muscle 22 alpha (SM22α) and alpha-smooth muscle actin (α-SMA) [2], and increased expression of bone-related proteins such as runt-related transcription factor 2 (Runx2) [3] and bone morphogenetic protein 2 (BMP-2) [4].

Vascular reactive oxygen species (ROS) alters in functional and structural vascular changes. Oxidative stress is reported to be an important mediator of osteochondrogenic trans-differentiation of VSMCs and is closely associated with the development of cardiovascular disease and vascular calcification in vitro and in vivo studies [3]. Tang et al. reported that hypercholesterolemia accelerated vascular calcification induced by excessive vitamin D via oxidative stress [5]. Moreover, hydrogen peroxide (H_2_O_2_) drives VSMC phenotypic from contractile to osteogenic type. The essential role of Runx2 in oxidative stress induced VSMC calcification was further confirmed by Runx2 depletion and overexpression. Taken together, accumulating evidence indicates that ROS and osteogenic transition are the important pathways in vascular calcification induced cardiovascular disease. Epidemiologic studies have demonstrated an association between consumption of plant-enriched diets and beverages and the improvement in some pathologies such as cardiovascular diseases [6]. These benefits have been often related to the presence of polyphenolic compounds and their anti-oxidative properties [7].

**Figure 1:**
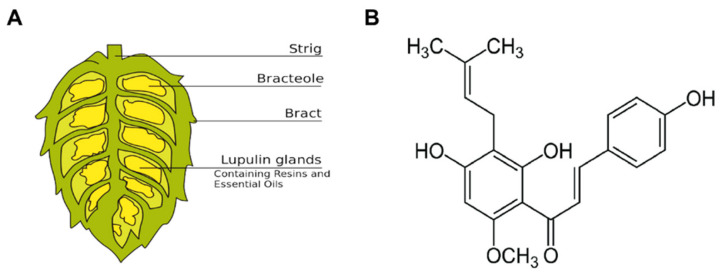
Xanthohumol (XN), a prenylated chalcone isolated from the hop plant. (**A**) Schematic drawing of a female hop cone that is composed of a central spine: the strig, bracteoles, bracts, and the characteristic lupulin glands that are tiny yellow sacs containing the hop oils. (**B**) Chemical structure of xanthohumol (3′-[3,3-dimethylallyl]-2′,4′,4-trihydroxy-6′-methoxychalcone).

Xanthohumol (2′,4′,4-trihydroxy-6′-methoxy-3′-prenylchalcone, XN), a structurally simple polyphenol chalcone that occurs only in hop plants (Figure 1). It has principal prenylated flavonoid in hops (up to 1% based on dry weight). Since the 1990s, interest in health-promoting activities of XN was constantly increased, scientific investigations were initialized worldwide and papers and patents on this topic have been steadily increasing [8]. XN is presented in micromolar concentrations in beers and exploited for preserving and flavoring beer, which has multiple biological activities including anticarcinogenic [8], anti-inflammatory, and antioxidant properties [9]. Yao et al. (2015) showed that XN activated nuclear factor erythroid 2-related factor 2 (Nrf2) enzymes to confer protection against oxidative damage in PC12 cells [10]. Moreover, XN is able to scavenge reactive oxygen species including hydroxyl and peroxyl radicals, and inhibits superoxide anion radical and nitric oxide production [11].

Nrf2 is a major regulator of the antioxidant defense system that mediates cell survival and regulates the gene expression encoding intracellular detoxifying enzymes and antioxidant proteins via antioxidant response element (ARE) [12]. Previous study reported that XN exerts anti-inflammatory activity through the transcription factor, Nrf2-ARE signaling in microglial cells, and suggested that it may be an attractive candidate to regulate the inflammatory responses in the brain [13].

Several studies have been shown that XN effects on health benefits such as anti-inflammatory, antioxidant, and anti-lipoperoxidative activities as well as antiangiogenic, antiproliferative, and apoptotic effects, both assessed in vitro and in vivo studies [14]. Regarding anti-inflammatory and antioxidant pathways, XN has the potential to reduce cholesterol accumulation in atherogenic plaque. Furthermore, XN is expected to be promising as an anti-atherosclerotic agent through its enhancement of lipoprotein metabolism. This means that XN has as a main function to inhibit atherosclerosis [15].

However, the molecular mechanism of XN against VC remains unclear. In this study, we investigated whether XN decreases oxidative stress generation against VC. XN inhibits osteogenic transition of VSMC through upregulation of Runx2 and BMP-2 and enhances its contractile phenotype via increased expression of α-SMA and SM22α. Furthermore, this study examined XN improves VC via the Nrf2/ Kelch-like ECH-associated protein 1 (Keap1)/ heme oxygenase-1 (HO-1) signaling pathway.

## 2. Materials and Methods

### 2.1. Experimental Animals

**Figure 2:**
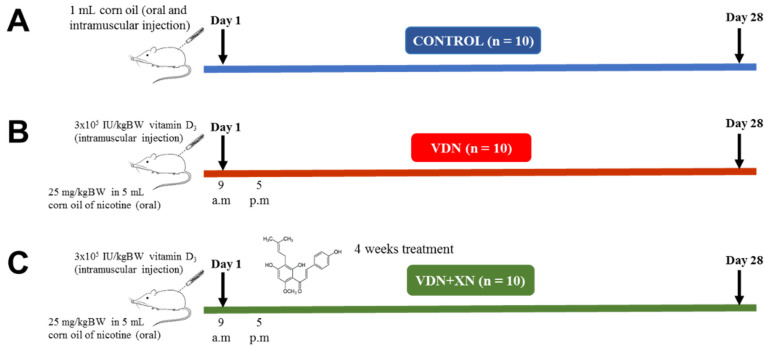
Outline of the experimental protocol. Sprague Dawley (SD) rats were randomly classified into three groups of 10 rats each. (**A**) Control group, (**B**) model (VDN) group, rats were intragastric administrated with 25 mg/kg BW in 5 mL corn oil of nicotine plus intramuscular injection of vitamin D_3_ (3 × 10^5^ IU/kg BW), and (**C**) treatment (VDN + XN) group, rats were orally treated with 20 mg/L in 0.1% ethanol for four weeks in drinking water.

As shown in Figure 2, 38-week-old male Sprague Dawley (SD) rats (180-200 g) were randomly divided into three groups: control group (CTL), model group (VDN group), and treatment group (VDN + XN). The preparation of VC model was made as previously described [14]. Briefly, the VDN and VDN + XN rats were intragastric administrated with 25 mg/kg BW in 5 mL corn oil of nicotine (Sigma Aldrich, Saint Louis, MO, USA) plus intramuscular injection of vitamin D_3_ (3 × 10^5^ IU/kg BW, Sigma Aldrich) at 9 a.m. on day 1. Afterward, nicotine administration was repeatedly administrated at 5 p.m. The VDN is a one-day only treatment. However, VDN + XN group was provided nicotine, vitamin D_3_, and XN (X741950, Toronto Research Chemicals, Toronto, Canada) as well. XN (20 mg/L in 0.1% ethanol) was orally administrated for four weeks in drinking water. Rats in the control group received both oral gavage and intramuscular injection of corn oil. During the treatment period, body weight (BW) and heart index (organ weight/body weight) of all animals were weekly recorded. Food and beverages were daily renewed and monitored. After 4 weeks of treatment, animals were sacrificed. For euthanasia, the rats were sacrificed by 30% urethane injection. The thoracic aorta tissues were frozen at −80 °C for molecular analyses or fixed in 10% neutral-buffered formalin, dehydrated and paraffin-embedded for histological assays. Three-micrometer-thick tissue sections were used for hematoxylin–eosin histological staining. Blood was also collected for biochemical analyses.

All rats were allowed free access to food and water *ad libitum* during the acclimatization and experimental period. The rats were kept in a good ventilated room at standard experimental condition with room temperature (22 ± 2 °C) and relative humidity (60 ± 10%) with a 12 hours light/dark cycle. SD rats were purchased from Bio LASCO Taiwan Co., Lt., Taiwan. The experiments were conducted in accordance with the ARRIVE (Animal Research: Reporting of in vivo Experiments) guidelines and approved by the Kaohsiung Medical University animal center (Kaohsiung, Taiwan, IACUC Approval No. 107060).

### 2.2. Measurement of Calcium Content in Aorta

The aorta tissue was homogenized and prepared in lysis buffer [20 mM Tris-HCl, 1 mM dithiothreitol (DTT), 5 mM EGTA, 0.5 mM PMSF, 20 μM leupeptin, and 20 μM aprotonin]. The calcium content in aorta was extracted and measured by a calcium assay kit (Bio Assay system, 3191 Corporate Place, Hayward, CA 94545, USA) according to the manufacturer’s instructions.

### 2.3. Measurement of ALP Activity in Aorta

The aorta tissue was homogenized and prepared in 1% Triton X-100. After centrifugation at 13,000 rpm for 20 min at 4 °C, the protein content in supernatant was tested. The activity of alkaline phosphatase (ALP) was measured by the ALP assay kit (Bio Assay system, 3191 Corporate Place, Hayward, CA 94545, USA) according to the manufacturer’s instructions.

### 2.4. ROS Detection

Tissues were removed, immersed in saccharose (30% *w/v*), embedded in optimal cutting temperature (OCT) and stored at −20 °C until immunofluorescence assay. OCT blocks were 10 μm cut in a cryostat and mounted on polylysine-coated glass slides. Tissue sections were incubated in 4 μM dihydroethidium (DHE; Thermo Fisher Scientific Inc., Waltham, MA, USA) or 10 μM 2′,7′-dichloroflurescin diacetate (DCFH-DA; Thermo Fisher Scientific Inc., USA) for 30 min at 37 °C in a humidified chamber protected from light. In the presence of superoxide anion, DHE is oxidized to ethidium that yields bright red fluorescence. After washing with PBS, sections were mounted and visualized by confocal laser-scanning microscope (Olympus Fluoview FV1000, Tokyo, Japan).

### 2.5. Histopathological Examination

Aorta tissue was fixed in 10% paraformaldehyde solution (Sigma-Aldrich, Saint Louis, MO, USA), followed by paraffin embedding. The embedded vascular tissue was cut into 5 μm sections. After dewaxing with xylene and hydration by passing a series of graded ethanol concentrations, hematoxylin (Sigma-Aldrich, Saint Louis, MO, USA) staining was performed for 2 min. After washing with tap water, aorta tissue sections were treated with eosin (Sigma-Aldrich, Saint Louis, MO, USA) staining for 3 min. Afterward, the sections were washed, dehydrated, and treated by xylene before microscopic observation. The aorta tissues were photographed under an optimal microscope (Nikon ECLIPSE TE2000-S, Tokyo, Japan).

### 2.6. Alizarin Red S Staining

For Alizarin Red S staining, aortic slides were dehydrated, rinsed rapidly in distilled water, and placed in an Alizarin Red S (Sigma-Aldrich, Saint Louis, MO, USA) staining solution for 10 min at room temperature. Any unbound stain was removed from the tissues, then photographed under an optimal microscope (Nikon ECLIPSE TE2000-S, Tokyo, Japan).

### 2.7. Immunohistochemistry Analysis

The aorta tissue in each group was embedded in paraffin, sliced, and dewaxed into water. Then, the aorta tissue was activated with Peroxidase Block for 10 min and blocked in 3% bovine serum albumin (BSA) for 30 min. The aorta tissue was incubated overnight at 4 °C with primary antibody BMP-2 (1:50, ARG57829, Arigo, Santa Cruz, CA, USA), Runx2 (1:50, sc-10758, Santa Cruz, Dallas, TX, USA), α-SMA (1:320, #2547, Sigma-Aldrich, Saint Louis, MO, USA), and SM-22 α (1:250, ab14106, ABCAM, Cambridge, MA, USA). Subsequently, horseradish peroxidase-labeled goat anti-rabbit IgG (1:100) was used. 3′-Diaminobenzidine (DAB) coloration, counterstaining, dehydration, clearing, and mounting were applied. The expressions of BMP-2, Runx2, α-SMA, and SM22α were observed under optical microscope (Nikon ECLIPSE TE2000-S, Tokyo, Japan). For image processing and analysis, the microscope was equipped with Nikon DS Fi1 digital camera and NIS-Element F software (Tokyo, Japan). Three fields were randomly selected from each slice.

### 2.8. Immunofluorescence Analysis

Tissues were removed, immersed in saccharose (30% *w/v*), embedded in OCT, and stored at −20 °C until immunofluorescence assay was performed. OCT blocks were cut in a cryostat and mounted on polylysine-coated glass slides. Ten μm thick sections were incubated with 10% paraformaldehyde solution. After washout in PBS plus 0.05% Triton X-100, the sections were blocked for 30 min with 5% BSA and then incubated with rabbit antibodies against Nrf2 (1:100 dilution; Proteintech Group, Inc., Rosemont, IL, USA), rabbit antibodies against Keap1 (1:100 dilution; Proteintech Group, Inc., Rosemont, IL, USA), and mouse antibodies against HO-1 (1:50 dilution; Proteintech Group, Inc., Rosemont, IL, USA) overnight at 4 °C. Then, the sections were incubated with a secondary Goat Anti-Rabbit IgG Antibody, (H+L) FITC conjugate (dilution 1:100; Sigma-Aldrich, Saint Louis, MO, USA), and nuclei were stained with 4, 6-diamidino-2-phenylindole (DAPI, Sigma-Aldrich, Saint Louis, MO, USA) at room temperature. Sections were mounted and viewed by confocal laser-scanning microscope. Five random images from each specimen were captured and area displaying fluorescence was quantified and normalized by total nuclei number by using Image J software. An average value for each specimen was obtained. The treatment corresponding to each specimen was blinded for the investigator capturing and quantifying immunofluorescence images.

### 2.9. Western Blotting Analysis

The aorta tissue was homogenized and centrifuged at 13,000 rpm for 30 min at 4 °C to take the supernatant. The extract of aorta tissues was prepared in lysis buffer [20 mM Tris-HCl, 1 mM dithiothreitol (DTT), 5 mM EGTA, 0.5 mM PMSF, 20 μM leupeptin, and 20 μM aprotonin]. Separation of total protein were done by using sodium dodecyl sulfate polyacrylamide gel electrophoresis (SDS-PAGE) on 10–14% acrylamide gels, then transferred to a polyvinylidine diflouride (PVDF). After blocking with 5% fat-free milk for 1 hour, the membranes were incubated overnight at 4 °C with the respective primary antibodies rabbit antibodies against SM22α (1:1000, ab14106 Abcam, Cambridge, MA, USA), α-SMA (1:1000, D4K9N, USA), BMP-2 (1:500, ab14933, Abcam, Cambridge, MA, USA), Runx2 (1:100, sc-390351, Santa Cruz, Dallas, TX, USA), Nrf2 (1:500, tcba 1560, Taiwan), Keap1 (1:1000, 10503-2-AP, USA), and HO-1 (1:250, sc-390991, Santa Cruz, Dallas, TX, USA). The blots were visualized with enhanced chemiluminescence (ECL) system (Millipore Corporation, Billerica, MA 01821 USA) and Image J software was used to scan and quantify the gray values.

### 2.10. Statistical Analysis

All continuous variables were expressed as mean ± standard error of mean (SEM). Bar charts represent mean values and error bar indicates SEM. All statistical analyses were performed using one-way analysis of variance (ANOVA) and followed by the Student’s *t*-test analysis. The data were analyzed using SigmaPlot version 12.0 software. A significant difference was determined at a *p*-value less than 0.05.

## 3. Results

### 3.1. Effects of XN Physiological Markers in VDN-Induced VC

**Figure 3:**
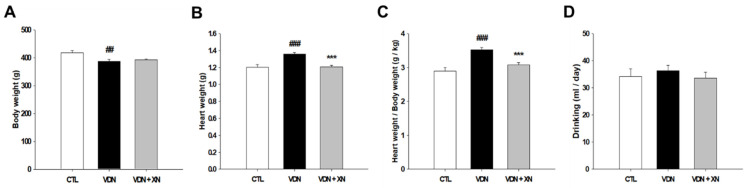
Effects of XN on physiological markers of VDN-induced VC rats. (**A**) Body weight, (**B**) heart weight, (**C**) heart body weight ratio, and (**D**) average daily drinking intake. Each point represents the mean ± SEM, *n* = 10. ^##^ *p* < 0.01, ^###^ *p* < 0.001 compared with the control group. *** *p* < 0.001 compared with VDN group.

Throughout the whole sequence of experiment, the rat mortality rate was zero. Afterward, to determine whether XN administration caused alteration in some physiological markers, we generated some observation such as body weight evaluation, heart body weight index calculation, and measurement of drinking consumption. At the end of study, rats’ body weight in the VDN group was lower than the other groups (*p* < 0.01), indicating that VDN administration caused a reduction in a rat’s body weight (Figure 3A). However, the heart-body weight ratio in the VDN group had a higher index than in the other groups (*p* < 0.001). Treatment with XN resulted in an improvement of the heart body weight ratio (*p* < 0.001, Figure 3C). This means that XN prevented cardiac enlargement in VC rat model. In addition, there were no significant differences among all groups in daily drinking consumption (Figure 3D).

### 3.2. Effects of XN on the Attenuation of Vascular Calcification Markers in VDN-Induced VC

**Figure 4:**
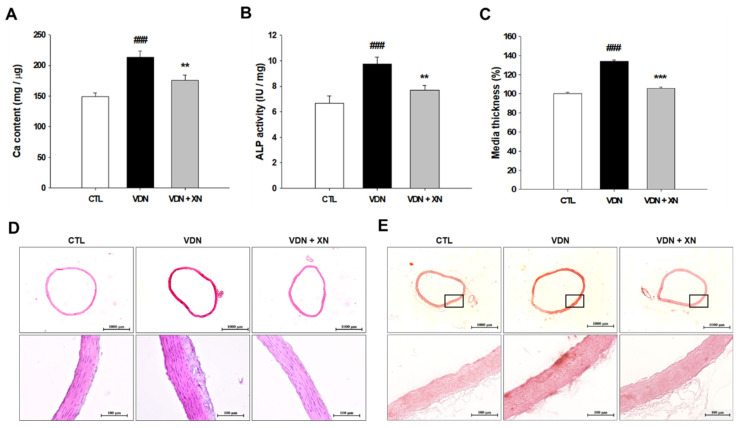
Effects of XN on aortic mineralization and osteoblastic phenotype. (**A**) Calcium content, (**B**) ALP activity, and (**C**) media thickness. Histopathological staining of rats’ aorta (**D**) hematoxylin and eosin staining and (**E**) Alizarin red S staining (20× and 200× magnification). Each point represents the mean ± SEM, *n* = 10. ^###^ *p* < 0.001 compared with the control group. ** *p* < 0.01, *** *p* < 0.001 compared with the VDN group.

To investigate whether XN ameliorates VDN-induced VC in rat’s aorta, we determined the calcium content and ALP activity. In the VDN group showed the highest aortic calcium content compared with other groups (*p* < 0.01, Figure 4A). Similarly, the ALP activity, a marker of osteoblast differentiation, was increased in VDN-induced VC rats compared with other groups (*p* < 0.01, Figure 4B). Treatment with XN significantly reduced the level of aortic calcium content and ALP activity in VDN-induced VC rats.

Furthermore, to evaluate aortic remodeling caused by VC, we generated histopathology staining in aorta. In VDN group, it showed that aortic medial layer was the thickest compared with other groups (*p* < 0.001, Figure 4C,D). Furthermore, Alizarin Red S staining was used to observe the effect of XN administration in VC-induced rat. Compared with control rats, VDN-induced VC in a rat’s aorta showed extensive calcification in the entire aortic wall. As presented in Figure 4E, XN treatment led in inhibition of rats’ calcification. The findings suggested that XN treatment prevents vascular medial thickening and calcification in VDN-induced rat VC.

### 3.3. Effects of XN in Inhibition of BMP-2 and Runx2 in VDN-Induced VC

**Figure 5:**
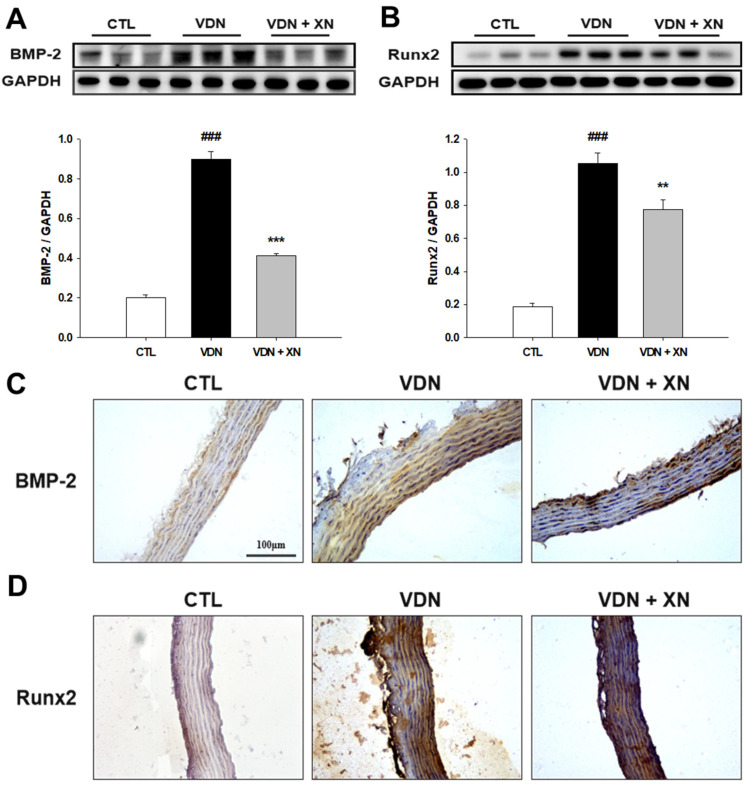
Effect of XN on osteoblastic-related protein levels. Western blot assay of (**A**) BMP-2 and (**B**) Runx2. GAPDH was a control for protein loading. Results were obtained from six independent experiments, and densitometric analysis of autoradiograms is shown as the ratio to GAPDH. Immunohistochemical staining to detect the expressions of (**C**) BMP-2 and (**D**) Runx2 (200× magnification). Each point represents the mean ± SEM, *n* = 10. ^###^ *p* < 0.001 compared with the control group. ** *p* < 0.01, *** *p* < 0.001 compared with VDN group.

Bone-associated factors are present during the progress of mineralization in vasculature. Thus, we further determined the expression of osteogenic marker proteins, including BMP-2 and Runx2 by western blot analysis. As shown in Figure 5A,B, compared with control group, VDN-induced rat VC caused an increase in both BMP-2 and Runx2 expressions (*p* < 0.001). The treatment of XN resulted in decreased expression of BMP-2 and Runx2 (*p* < 0.01). Additionally, immunohistochemical staining of both protein expressions showed similar findings as the western blot assay. As presented in Figure 5C,D, compared with control group, VDN-induced rat VC affected the marking of BMP-2 and Runx2 expressions. Furthermore, treatment with XN reduced the expression of both these proteins. These assays showed that XN significantly downregulates the osteoblast-like phenotype markers, BMP-2 and Runx2 expressions.

### 3.4. Effects of XN on the Expressions of α-SMA and SM22α in VDN-Induced VC

**Figure 6:**
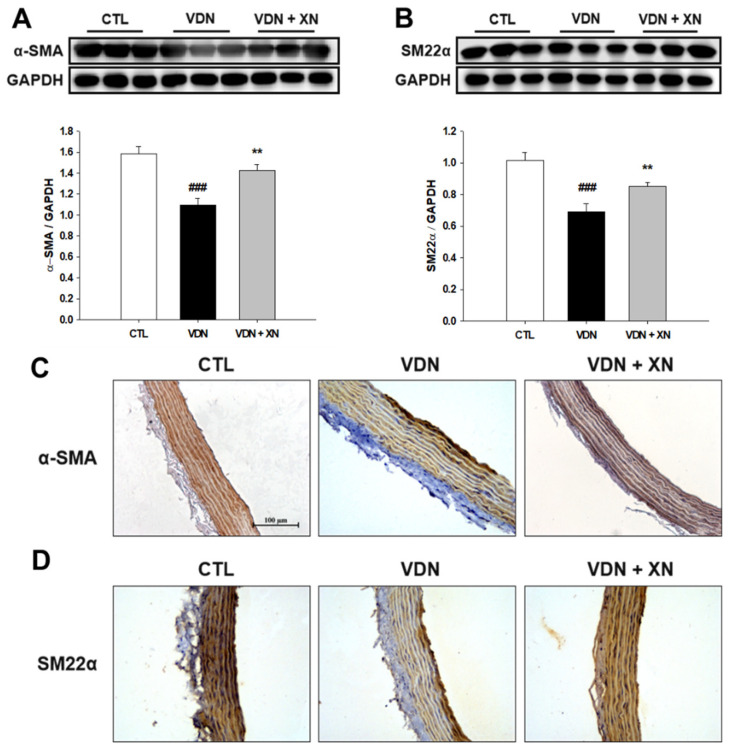
Effect of XN on vascular contractile phenotype markers. Western blot assay of (**A**) α-SMA and (**B**) SM22α. GAPDH was a control for protein loading. Results were obtained from six independent experiments, and densitometric analysis of autoradiograms is shown as ratio to GAPDH. Immunohistochemical staining to detect the expressions of (**C**) α-SMA and (**D**) α-SMA (200× magnification). Each point represents the mean ± SEM, *n* = 10. ^###^ *p* < 0.001 compared with the control group. ** *p* < 0.01 compared with VDN group.

Phenotype transition of VSMCs is associated with VC in vivo. Therefore, the expression of VSMCs markers such as α-SMA and SM22α were investigated. As shown in Figure 6, compared with the control group, both α-SMA and SM22α expressions were decreased in VDN-induced rat VC (*p* < 0.001). The administration of XN significantly increased the expressions of these contractile phenotype related proteins (*p* < 0.01).

Similar to immunoblotting results, as presented in Figure 6C,D, the immunohistochemical staining showed that VDN-induced rat VC had spotted expressions of both α-SMA and SM22α in the aortic walls. Moreover, control and XN treatment groups showed less expression of these vascular phenotype markers.

### 3.5. Effects of XN in Reduction of Oxidative Stress Markers in VDN-Induced VC

**Figure 7:**
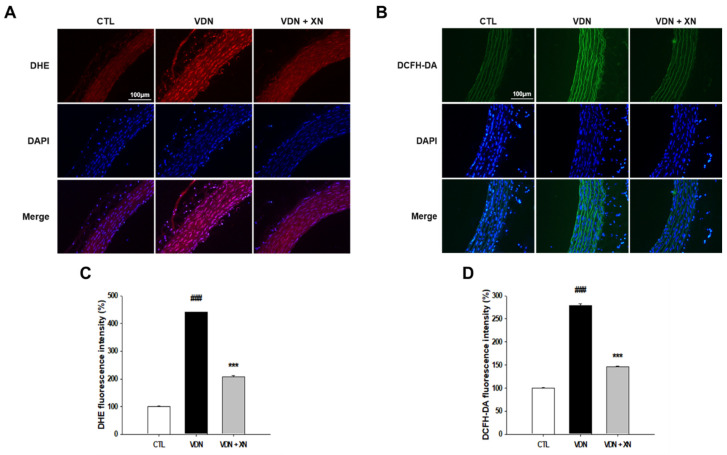
Effects of XN on oxidative stress markers through confocal microscopic observation of nuclear thoracic aorta wall. (**A**) DHE staining to detect anion superoxide generation, (**B**) DCFH-DA staining to identify H_2_O_2_ production (magnification 200x), (**C**) quantified DHE fluorescence intensity, and (**D**) quantified DCFH-DA fluorescence intensity, relative to number of nuclei. Counterstaining of nuclei with DAPI in blue was merged into all images. Each point represents the mean ± SEM, *n* = 6. ^###^ *p* < 0.001 compared with the control group. *** *p* < 0.001 compared with VDN group.

To investigate whether XN blocks the in situ oxidative stress indicators, we measured aortic superoxide generation as detected by DHE staining and H_2_O_2_ production through DCFH-DA staining. As shown in Figure 7, superoxide generation in thoracic aorta was 17.6 ± 2.54 a.u (arbitrary units), 84.25 ± 1.79 a.u, and 36.81 ± 5.19 a.u, respectively, in CTL, VDN, and VDN + XN. VDN had significantly increased DHE fluorescence level compared to the control group (*p* < 0.001). XN treatment inhibited superoxide production in VDN-induced rat VC (*p* < 0.001). The DCFH-DA staining in thoracic aorta was 25.19 ± 1.54 a.u, 70.41 ± 3.79 a.u, and 36.77 ± 1.89 a.u, respectively, in CTL, VDN, and VDN + XN. VDN had significantly increased the DCFH-DA fluorescence level compared to the control group (*p* < 0.001). XN treatment inhibited hydrogen peroxide generation in VDN-induced rat VC (*p* < 0.001). These results suggested that XN inhibits oxidative stress VDN-induced rat VC.

### 3.6. Effects of XN on the Expressions of Nrf2, HO-1, and Keap1

**Figure 8:**
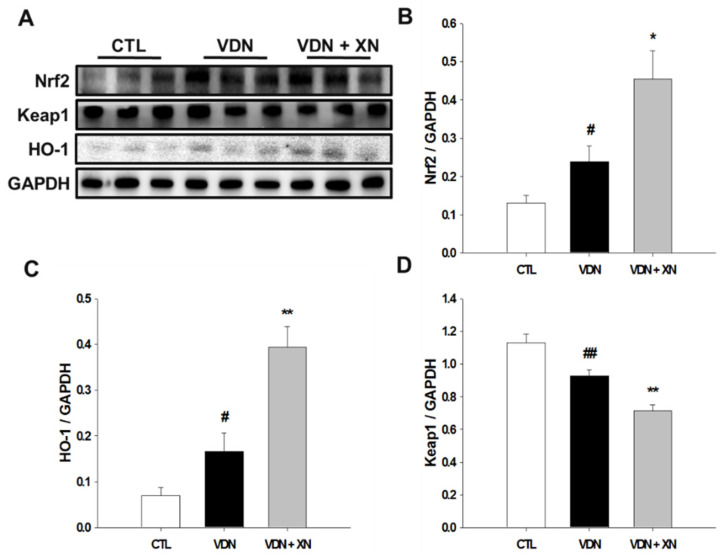
(**A**) Effects of XN treatment on protein levels of (**B**) Nrf2, (**C**) HO-1, and (**D**) Keap1 through western blot analysis. GAPDH was a control for protein loading. Results were obtained from six independent experiments, and densitometric analysis of autoradiograms is shown as ratio to GAPDH. Each point represents the mean ± SEM, *n* = 10. ^#^ *p* < 0.05, ^##^ *p* < 0.01 compared with the control group. * *p* < 0.05, ** *p* < 0.01 compared with VDN group.

**Figure 9:**
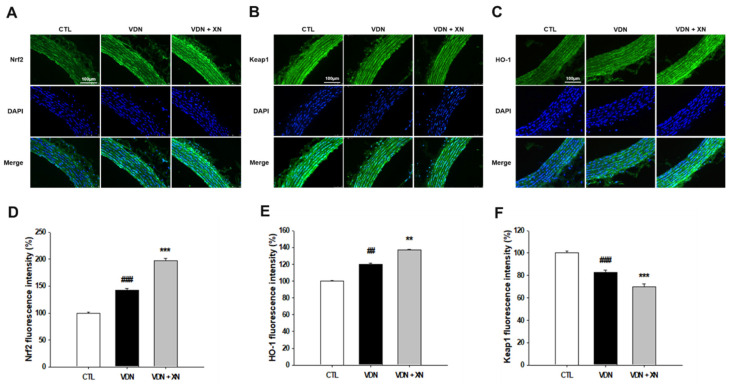
Effects of XN on the expressions of Nrf2, HO-1, and Keap1 through confocal microscopic evaluation of nuclear thoracic aorta wall. (**A**) Nrf2, (**B**) HO-1, (**C**) Keap1, (**D**) quantified Nrf2 fluorescence intensity, (**E**) quantified HO-1 fluorescence intensity, and (**F**) quantified Keap1 fluorescence intensity, relative to number of nuclei. Counterstaining of nuclei with DAPI in blue was merged into all images. Each point represents the mean ± SEM, *n* = 6. ^##^ *p* < 0.01, ^###^ *p* < 0.001 compared with the control group. ** *p* < 0.01, *** *p* < 0.001 compared with VDN group.

We observed XN treatment on VDN-induced rat VC through the activation of Nrf2, a main critical transcription factor regulates antioxidant responses. Nrf2 is an important antioxidant signal involved in vascular calcification. As shown in Figure 8, western blot analysis showed that VDN administration significantly upregulated both the expression of Nrf2 and its specific target gene HO-1 in comparison with the control group (*p* < 0.05). Compared to the model group, XN treatment significantly reduced these protein expressions (*p* < 0.05). In the same way, fluorescence observation showed increased expression of these proteins in control and XN treatment as well (Figure 9D,E). Contrarily, the expression of Keap1 was significantly decreased in VDN group compared with control group (*p* < 0.01, Figure 9F). In comparison with control and model group, XN treatment group had the lowest expression of Keap1 (*p* < 0.05). Fluorescence observation indicated similar results.

## 4. Discussion

The continuing increase in the prevalence of hypertension, atherosclerosis, and diabetes in the general population is predicted to result in a higher incidence of cardiovascular and kidney diseases. There is an appreciation of increased disease burden and higher levels of vascular calcification found in the patients who suffered from these diseases. VC has inseparable relationship with pathomorphological condition in vascular structure and function. VC driven vascular stiffness leads to functional disturbance and it is a main predictor of cardiovascular mortality. Loss of intimal or medial layer in calcification-related vascular wall caused oxidative stress, endothelial dysfunction, increased inflammatory cytokine production, alterations in mineral metabolism, and release of osteoprogenitor cells from the marrow into the circulation. Therefore, for the advance of novel treatments particularly targeting vascular calcification, a serious concern of the implications for oxidative stress and bone metabolism must be taken into account to avoid potentially harmful effects to cardiovascular health.

In the present study, we generated VC through in vivo study by the administration of vitamin D_3_ plus nicotine on SD rats. As previous study, VDN resulted in induction of calcium content and ALP activity overload on the arteries and media calcification, which are analogue with human atherosclerosis and arteriosclerosis [16]. In this study, treatment with vitamin D_3_ plus nicotine for four weeks effected in increased of calcium content and ALP activity in the aortic tissues. Moreover, it leads to the thickened vessel walls, disorder of elastic fibers and a marked calcification area in aorta wall. These changes caused by vascular calcification were in accordance with previous research [17]. In our research, we found that treatment with XN exerted protective effects on VC. XN administration inhibited the increase of ALP activity and calcium-phosphate salt deposition in calcified aortas. Meanwhile, histopathological evaluation also showed the amelioration consequence of XN treatment on the structural damage of calcified vessel walls. These findings provide suggestion that XN has potent preventive effects on arterial calcification.

VC is also reflected by osteochondrogenic differentiation markers including Runx2 and BMP-2, and inhibition of smooth muscle cell lineage markers α-SMA and SM22α [18]. Runx2 expression has been identified in atherosclerotic calcified of human vascular tissue specimens [19] and in calcifying aortic smooth muscle cells in mice [20,21] but not in normal vessels. Furthermore, increased expression of Runx2 is associated with VSMC calcification in vitro [22], supporting a role for Runx2 in VC. BMP-2, a member of the transforming growth factor beta (TGF-β superfamily), has been identified as one of the most important molecules to regulate bone formation and osteoblast differentiation [23]. Previous studies have demonstrated that BMP-2 regulates osteoblast differentiation through osteogenic transcription factors including Runx2 and Msx2 [24,25]. Several studies have revealed that BMP-2 positively regulates vascular calcification [26,27]. The inhibition of BMP signaling reduces vascular calcification [28]. Runx2 is a major target protein of the BMP pathway, which is a regulator of osteogenesis and is crucial for regulating the expression of bone specific genes. However, BMP2 signaling stimulates p300- mediated Runx2 acetylation, thereby increasing transactivation activity and inhibiting Smurf1-mediated degradation of Runx2 [29]. Therefore, we further determined the expression of osteogenic marker proteins including Runx2 and BMP-2. In this study, VDN-induced rat VC led to increase the protein levels of Runx2 and BMP-2 and treatment with XN ameliorated these protein expressions. Moreover, the inhibitory effect of XN on Runx2 and BMP-2 expressions were further confirmed by immunohistochemical staining. These findings suggest that XN effects in the inhibition of bone-associated factors in the progress of mineralization in vasculature.

Our results showed that the protein levels of vascular contractile phenotype markers SM22α and α-SMA were significantly decreased in VDN group compared with CTL group. XN treatment improved the expressions of SM22α and α-SMA. Similarly, immunohistochemical staining showed downregulation of SM22α and α-SMA expressions in VDN group. Moreover, treatment with XN upregulated these protein expressions. These results indicated the therapeutic effects of XN on VC was attributed by its inhibiting effect on VSMC phenotypic remodeling.

Accumulating evidences have implicated ROS-induced oxidative stress in the progression of vascular calcification [30,31]. In the present study, we used an in vivo model of VSMC calcification induced by vitamin D_3_ plus nicotine to reveal the increased intracellular ROS production. It is consistent with our findings that VC is linked to oxidative stress [32]. Increased ROS production that exceeds the capacity of cellular antioxidant mechanisms is considered to be a fundamental cause of the osteogenic differentiation of VSMCs, leading to vascular calcification [3]. The precise mechanism involved in the induction of oxidative stress by vitamin D_3_ plus nicotine is still unclear. However, there is evidence that oxidative stress is a major driver of calcification in atherosclerosis and chronic kidney disease. Some studies have suggested that oxidative stress plays a critical role in the pathogenesis of VC, and it is associated with certain conditions such as hypercholesterolemia, hypertension, diabetes mellitus, and dialysis-dependent end stage renal disease [33]. A brief summary of the existing literature regarding antioxidants for VC is important, as no effective treatment is currently obtainable for this disease [34,35,36,37]. Therefore, it is important to increase antioxidative capacity and reduce oxidative to prevent VC. This study provided potential findings that XN reduced the superoxide and hydrogen peroxide generations in VDN-induced vascular calcification in rats.

XN has been shown to ameliorate an excessive oxidative stress in VC by upregulating Nrf2, a critical redox-sensitive transcription factor. It is activated to improve the oxidative stress state of the body, promote cell survival, and maintain cells redox homeostasis by regulating the induced expression of phase-II detoxifying enzymes and antioxidant enzymes [38]. Nrf2, which is suppressed by the negative regulator Keap1, protects against oxidative stress by inducing the expression of antioxidant proteins and phase 2 detoxification enzymes in response to changes in the intracellular redox balance. When cells are attacked by ROS or electrophiles, Nrf2 dissociates from Keap1 and is rapidly transferred to the nucleus. Phosphorylated Nrf2 forms a heterodimer with Maf protein and then combines with AREs, which activate the expression of HO-1 [39]. HO-1 is an important endogenous antioxidant and constitutes an important defense system. Previous study showed that HO-1, one of the Nrf2 target genes, promotes autophagy and eliminates damaged mitochondria so as to repress oxidative stress in lipopolysaccharide treated rat liver [40,41]. According to the results of previous studies, an imbalance of oxidant-antioxidant system caused by increased ROS production may result in cell death. Nrf2 decreases intracellular ROS levels through its antioxidant activity [42,43]. In the present study, vascular calcification induced by vitamin D_3_ plus nicotine resulted in the upregulation of the transcription of Nrf2 and its nuclear translocation. Additionally, the level of Nrf2 target gene HO-1 was also upregulated. These results indicated that vitamin D_3_ plus nicotine may stimulate antioxidant activity by increasing Nrf2 expression and promote its nuclear translocation. However, mild oxidative stress can activate a protective mechanism via the Nrf2 signaling pathway, while high ROS accumulation activates a stress signal related to cell death rather than survival [44]. Additionally, according to the previous report, Nrf2 signaling is activated in response to ROS and regulates bone metabolism positively or negatively depending on the degree of oxidative stress [45]. Nrf2 signaling positively controls bone homeostasis by maintaining an intracellular redox balance, whereas Nrf2 may negatively regulate cellular differentiation to osteoblasts through inhibition of the Runx2-dependent transcriptional activity. A previous study, sharing the same conclusion, also indicated that the overexpression of Nrf2 binding to ARE could influence the process of cellular differentiation by interfering with Runx2 and its downstream targets [46]. Furthermore, Nrf2 was shown to inhibit osteoblast differentiation and mineralization via the suppression of some key regulatory proteins, such as Runx2. This is relevant in smooth muscle cells, where Runx2 deficiency inhibits VC occurrence [20,47].

Xanthohumol is widely used as a raw material in the brewing industry, to preserve beer and to give beer its characteristic aroma and flavor. In addition to their application in the brewing industry, hops have for a long time been used for various medical purposes. [48]. Recent studies have been shown that XN has potential functions for therapeutic purposes such as for prevention of type-2 diabetes, osteoporosis, coronary heart disease, neurodegenerative disorders (dementia, Alzheimer, Parkinson), hypertension, atherosclerosis, and many types of cancer [14]. Shati (2017) showed that XN prevented renal damage caused by ischaemia reperfusion injury through in vivo study [49]. Li et al. (2018) showed that XN significantly attenuated cisplatin induced nephrotoxicity. XN protects renal damage through the inhibition of inflammatory response and oxidative stress [50].

We summarized that antioxidant properties of XN treatment has contributing factors for its beneficial effects in vascular calcification as well as cardiovascular disease. Our findings proposed that XN has an inhibition of osteogenic transition in VDN rats through the downregulation of Runx2 and BMP-2 expressions and upregulation of the contractile phenotype α-SMA and SM22α expressions. Administration of XN attenuated the calcium content and ALP activity in calcified aortas. Moreover, XN also decreased the ROS, which then lead to Nrf2 activation and translocation. Our research confirmed that XN serves as an antioxidant factor against vascular calcification caused by oxidative stress and the osteogenic transition pathway.

## 5. Conclusions

It can be suggested through this in vivo study that XN has the potential to improve antioxidant capacity by regulating the Nrf2/Keap1/HO-1 pathway and further prevent VC. Bone-related factors were downregulated by treatment of XN. Otherwise, XN treatment leads to enhance vascular contractile phenotype markers SM22α and α-SMA. This study showed that XN may have the important effects to decrease cardiovascular risk by reducing VC. Nonetheless, advanced experimental studies are required to better understand the underlying mechanisms of potential effects of XN.
